# Gender-specific correlations between remnant cholesterol and severe abdominal aortic calcification in American adults

**DOI:** 10.3389/fendo.2024.1415424

**Published:** 2024-09-16

**Authors:** Laisha Yan, Xiaoyan Hu, Shanshan Wu, Shunying Zhao

**Affiliations:** Department of Cardiosurgery Intensive Care Unit, Ningbo Medical Centre Li Huili Hospital, Ningbo, China

**Keywords:** remnant cholesterol, cholesterol, gender differences, NHANES, abdominal aortic calcification

## Abstract

**Background:**

Remnant cholesterol (RC) predicts cardiovascular risk and is associated with a range of diseases, including asthma, hypertension, depression, periodontitis, and alcoholic fatty liver disease. However, its correlation with abdominal aortic calcification (AAC) has not been reported.

**Methods:**

Using a cross-sectional approach, this study examined data from the 2013-2014 National Health and Nutrition Examination Survey (NHANES) cycle. Multiple logistic regression, generalized summation models, and subgroup analyses were used in examining the correlation between RC and the prevalence of severe AAC.

**Results:**

The mean age of participants in this study was 57.70 ± 11.73 years, with 142 individuals (9.67%) suffering from severe AAC. The median RC was 0.52 mmol/L (Q1-Q3, 0.36-0.75 mmol/L). Among female participants, a significant positive correlation was observed between RC and severe AAC (per natural log [RC] increment: 2.14; 95% CI, 1.07-4.27). Smooth curve fitting and threshold effect analysis revealed a saturation effect at an RC level of 0.57 mmol/L. Conversely, in male participants, no significant correlation was found between RC and the prevalence of severe AAC (per natural log [RC] increment: 0.88; 95% CI, 0.43-1.78). Our findings suggest a significant interaction between gender and RC in relation to severe AAC (*P* for interaction = 0.0042).

**Conclusions:**

Higher RC levels were significantly associated with an increased prevalence of severe AAC in women.

## Background

Calcification typically first manifests in the aorta, and its prevalence increases with age (1). The Framingham Heart Study revealed that fewer than one-sixth of participants aged 45 displayed detectable signs of abdominal aortic calcification (AAC). By the age of 65, the prevalence of AAC increased to approximately 90% (2). The MESA (Multi-Ethnic Study of Atherosclerosis) study further showed that AAC prevalence varied from 34% in the 45-54 age group to 94% in those aged 75-84 (3). AAC serves as a potential indicator of subclinical atherosclerosis and is associated with a heightened relative risk of various outcomes, including coronary and cerebrovascular events, overall cardiovascular incidents, and cardiovascular-related mortality (4, 5). Its association with overall mortality is more pronounced than its association with coronary artery calcification (CAC) (6). Patients with severe AAC exhibit significantly higher absolute and relative risks for cardiovascular events, fatal cardiovascular incidents, and all-cause mortality compared to those with no or minimal AAC (7).

Remnant cholesterol (RC) encompasses the cholesterol found in triglyceride-rich lipoproteins such as chylomicron remnants, as well as in very-low-density lipoproteins (VLDL) and intermediate-density lipoproteins (IDL) (8). Its potential value in predicting cardiovascular risk is garnering attention, positioning it as a new therapeutic target in the era of “beyond low-density lipoprotein cholesterol (LDL-C)” (9). Moreover, RC is linked to a variety of diseases, including asthma, hypertension, depression, periodontitis, and non-alcoholic fatty liver disease (NAFLD) (10–15). However, its correlation with AAC remains unreported.

Data from the 2013 to 2014 National Health and Nutrition Examination Survey (NHANES), encompassing a wide range of American adults, were analyzed to study the relationship between RC and severe AAC. This analysis also includes an exploration of potential modifying factors that may influence the relationship between RC and severe AAC.

## Methods

### Study population

**Figure 1:**
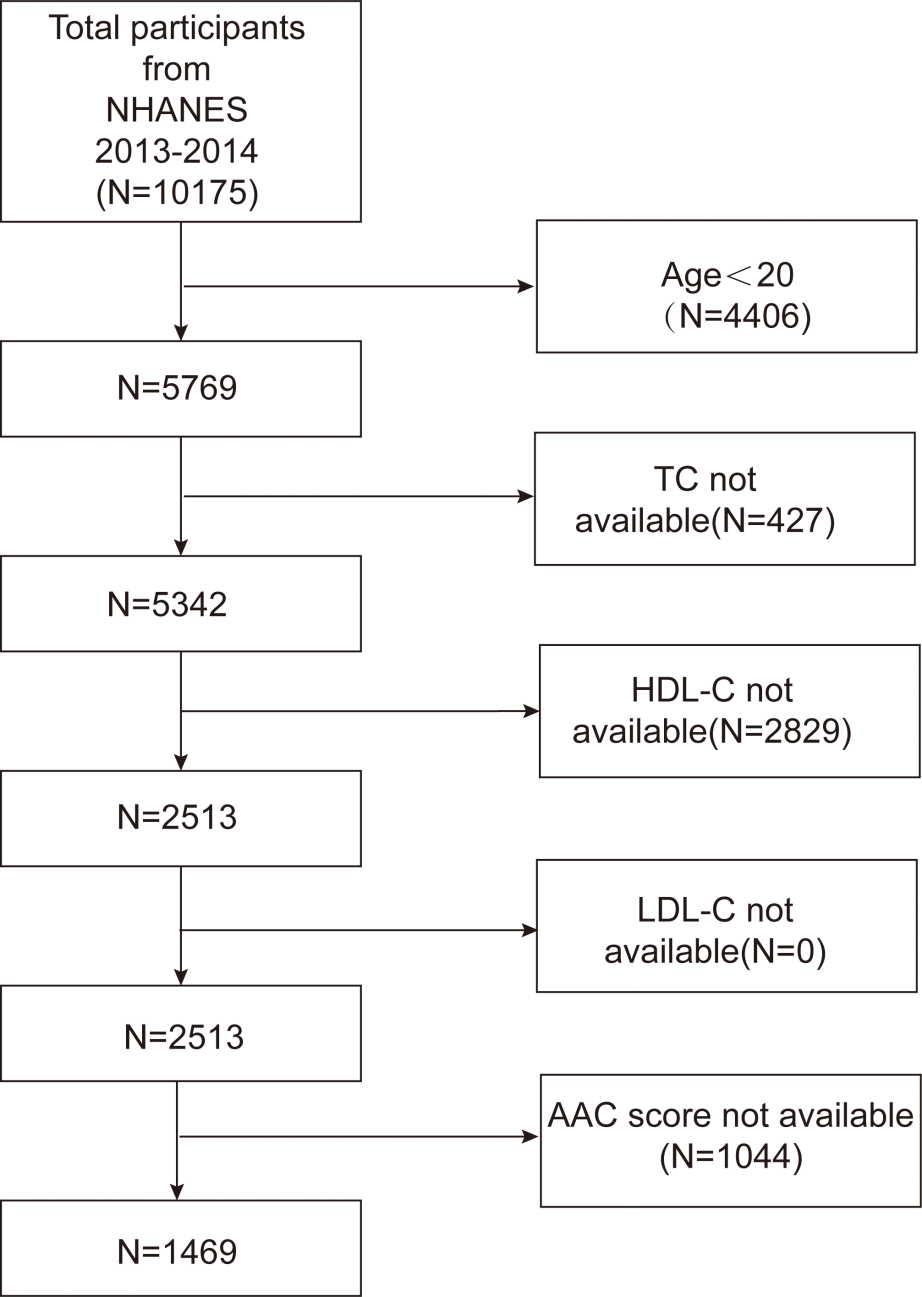
Flowchart of participant selection. TC, total cholesterol; HDL-C, high-density lipoprotein cholesterol; LDL-C, low-density lipoprotein cholesterol; AAC, abdominal aortic calcification.

The NHANES serves as a comprehensive database that captures the health and nutritional status of both children and adults in the United States. All participants provided informed consent, and the program was approved by an ethical review board. The present study analyzed data from the 2013-2014 cycle, including a total of 10,175 participants. Initially, subjects younger than 20 years of age were excluded from the study. In addition, participants lacking data on high-density lipoprotein cholesterol (HDL-C), total cholesterol (TC), and LDL-C, or without AAC score measurements, were also excluded. Consequently, 1,469 subjects remained eligible for analysis. **Figure 1** outlines the study’s inclusion and exclusion criteria.

### Study variables

The variable of interest, RC, is calculated using the formula RC = TC - LDL-C - HDL-C.

Severe AAC was defined as the outcome variable. Individuals aged 40 and above underwent dual-energy X-ray absorptiometry (DXA) scans. Participants were excluded based on the following criteria (1): pregnancy (2); recent exposure to radiographic contrast agents (barium) within the preceding 7 days (3); weight greater than 450 pounds; or (4) scoliosis with Harrington rods in the spine. Severe AAC was diagnosed when the Kauppila score exceeded 6.

Covariates were included in the final multivariate logistic regression models as potential confounders if they altered the RC estimates for severe AAC by more than 10% or were recognized as traditional risk factors for AAC. The included covariates encompassed various demographic, health, and biochemical indicators, including sex, age, race, education level, body mass index (BMI), presence of hypertension or diabetes mellitus, alcohol use, smoking status, and levels of creatinine (Cr), alanine aminotransferase (ALT), aspartate aminotransferase (AST), HDL-C, 25-hydroxyvitamin D (25[OH]D), and uric acid (UA). Hypertension status was determined by questionnaire. Diabetes mellitus diagnosis was based on one or more of the following criteria: determination through a questionnaire, fasting plasma glucose (FPG) levels of 7.0 mmol/L or higher, random glucose levels exceeding 11.1 mmol/L, HbA1c above 6.5%, or a 2-hour oral glucose tolerance test (OGTT) showing glucose levels of 11.1 mmol/L or more. BMI was classified into three categories: under 25 kg/m² as normal weight, 25-29.9 kg/m² as overweight, and 30 kg/m² or higher as obese. Smoking was defined as having smoked more than 100 cigarettes in a lifetime, and alcohol consumption was defined as consuming more than 12 drinks per year.

### Statistical analysis

Statistical analyses followed CDC guidelines. Sample weights were applied to account for the complex probability sampling of NHANES and to ensure representative adequacy, especially considering the oversampling in survey cycles.

The data were transformed to natural logarithms due to the non-normal distribution of RC. Descriptive analyses employed weighted t-tests and chi-square tests to evaluate differences among participants stratified by RC tertiles. Multiple logistic regression analyses were conducted to investigate the association between RC and severe AAC. We developed three analytical models: Model 1, which remained unadjusted; Model 2, which included adjustments for gender (only for the total population), age, and race; and Model 3, which incorporated corrections for all the previously mentioned covariates. To improve the robustness of the analysis, a sensitivity analysis was conducted by converting RC into categorical variables. The median value of each category of the independent variable was included in the model as a continuous variable to determine linear trends, and subsequent subgroup analyses were conducted. To detect any potential nonlinear associations, a generalized additive model was used, supplemented by smoothed curve fitting. Significant trend deviations were identified at inflection points using a two-stage linear regression approach. Statistical significance was defined as *P* < 0.05. All analyses and visualizations were performed using R (version 4.2.0) and EmpowerStats (version 4.0).

## Results

### Subject characteristics

The demographic and main clinical characteristics of the study population are summarized as follows. The mean age of participants was 57.70 ± 11.73 years. Among the participants, 142 individuals (9.67%) had a diagnosis of severe AAC. The overall sample consisted of 707 males (48.22%) and 762 females (51.78%). Of the participants, 6.88% were Mexican American, 4.62% were other Hispanic, 71.75% were non-Hispanic White, 9.58% were non-Hispanic Black, and 7.18% were from other racial groups. The mean age and prevalence of severe AAC in male participants were 57.37 ± 11.62 years and 9.48%, respectively. In female participants, the mean age and prevalence of severe AAC were 58.02 ± 11.82 years and 9.84%, respectively.

**Table 1** presents the demographic data of the study population according to tertiles of RC among male and female participants in the 2013-2014 NHANES. In males, higher RC was more common in younger adults, smokers, individuals with hypertension, diabetes, higher education levels, and Mexican Americans; BMI, UA, and ALT levels were also higher. Female participants with moderate to high RC were more likely to be older, have hypertension, diabetes, severe AAC, to be smokers, and to be Mexican Americans. They also had higher levels of BMI, UA, AST, and ALT compared to participants with low RC.

**Table 1:** Baseline characteristics of study participants.

Characteristics	Males			*P* value	Females			*P* value
	RC (mmol/L) tertiles				RC (mmol/L) tertiles			
	Tertiles 1	Tertile 2	Tertile 3		Tertiles 1	Tertile 2	Tertile 3	
RC range	< 0.42	0.42–0.65	> 0.65		< 0.39	0.39–0.63	> 0.63	
N	235	236	236		252	256	254	
Age, years	58.64 ± 11.62	57.73 ± 11.56	55.88 ± 11.51	0.0266	56.01 ± 11.95	58.22 ± 11.97	59.87 ± 11.21	0.0010
Race				0.0010				0.0112
Mexican American, %	2.86	10.56	7.40		5.32	7.27	8.28	
Other Hispanic, %	2.51	5.69	3.69		3.97	5.28	6.65	
Non-Hispanic White, %	75.58	67.29	74.80		68.41	69.63	74.27	
Non-Hispanic Black, %	14.21	9.05	5.67		14.16	10.88	3.65	
Other races, %	4.84	7.41	8.45		8.14	6.94	7.14	
Education level				0.0118				0.0600
Less than 9th grade, %	4	7.03	5.46		3.91	4.42	5.67	
9-11th grade, %	11.62	8.45	12.22		8.88	13.05	15.90	
High school graduate, %	25.62	18.65	18.10		17.15	22.69	20.97	
Some college or Associate of Arts degree, %	18.81	32.34	31.55		34.36	34.68	32.54	
College graduate or above, %	39.96	33.54	32.67		35.69	25.17	24.93	
BMI, kg/m^2^	26.09 ± 3.82	27.55 ± 3.80	29.78 ± 4.53	< 0.0001	27.11 ± 6.70	28.61 ± 5.87	30.60 ± 6.02	< 0.0001
HDL-C, mmol/L	1.55 ± 0.51	1.29 ± 0.27	1.08 ± 0.24	< 0.0001	1.85 ± 0.47	1.58 ± 0.39	1.31 ± 0.30	< 0.0001
TC, mmol/L	4.52 ± 0.82	4.93 ± 1.06	5.19 ± 1.08	< 0.0001	4.86 ± 0.93	5.12 ± 0.99	5.63 ± 1.07	< 0.0001
LDL-C, mmol/L	2.66 ± 0.74	3.10 ± 0.97	3.10 ± 0.97	< 0.0001	2.72 ± 0.76	3.03 ± 0.86	3.37 ± 1.06	< 0.0001
TG, mmol/L	0.67 ± 0.16	1.17 ± 0.15	2.33 ± 0.73	< 0.0001	0.63 ± 0.15	1.11 ± 0.14	2.07 ± 0.63	< 0.0001
Cr, mmol/L	91.78 ± 73.79	92.76 ± 37.96	91.87± 30.17	0.9757	70.60 ± 31.06	70.10 ± 17.13	68.41 ± 17.31	0.5299
UA, umol/L	341.01 ± 78.08	358.88 ± 68.35	383.30 ± 79.72	< 0.0001	269.54 ± 68.48	286.64 ± 65.82	318.09 ± 80.33	< 0.0001
AST, U/L	27.16 ± 14.81	24.83 ± 8.52	26.29 ± 10.71	0.1083	22.83 ± 9.74	22.52 ± 8.14	27.22 ± 30.72	0.0189
ALT, U/L	24.98 ± 15.67	25.71 ± 11.16	29.59 ± 16.37	0.0011	20.75 ± 12.00	20.82 ± 10.38	24.20 ± 15.47	0.0028
25(OH)D, nmol/L	58.86 ± 24.98	70.70 ± 24.14	68.51 ± 20.06	0.5585	84.58 ± 36.00	81.26 ± 32.31	77.72 ± 29.72	0.0621
Diabetes, %	16.72	17.21	30.15	0.0002	7.07	19.13	23.82	< 0.0001
Hypertension, %	33.42	34.12	47.36	0.0017	39.26	49.46	60.96	< 0.0001
Drinkers, %	86.02	90.15	85.85	0.3195	70.61	61.89	64.83	0.1199
Smokers, %	48.36	57.67	58.56	0.0461	33.27	38.20	46.12	< 0.0001
Severe AAC, %	8.31	8.25	5.68	0.4433	3.11	11.68	13.50	< 0.0001

RC, remnant cholesterol; BMI, body mass index; UA, uric acid; AAC, abdominal aortic calcification; Cr, creatinine; 25(OH)D, 25-hydroxyvitamin D; AST, Aspartate aminotransferase; ALT, Alanine aminotransferase; TG, triglyceride; LDL-C, low-density lipoprotein cholesterol; TC, total cholesterol; HDL-C, high-density lipoprotein cholesterol.

No significant differences were observed in alcohol intake, Cr, or 25(OH)D levels across the three RC tertiles in both male and female participants. Additionally, there were no statistically significant differences in AST levels or severe AAC among the three groups of male participants, nor in education levels among the three groups of female participants.

### Association between RC and severe AAC

The detailed outcomes of the multivariate analysis are presented in **Table 2**. In female patients, there was a significant positive correlation between RC and severe AAC (per natural log [RC] increment: OR, 2.14; 95% CI 1.07-4.27). Upon categorizing continuous RC into tertiles, the adjusted ORs for the second and third tertiles were 3.14 (95% CI: 1.32-7.46) and 3.17 (95% CI: 1.25-8.04), respectively, compared to the first tertile (*P* for trend <0.05). However, no association between RC and severe AAC was found in the overall population (OR: 1.33; 95% CI: 0.83-2.13) or in male participants (OR: 0.88; 95% CI: 0.43-1.78). Gender influenced the association between RC and severe AAC (*P* for interaction = 0.0042).

**Table 2:** Association between RC and severe AAC.

RC, mmol/L	Severe AAC OR (95% CI), *P* value		
	Model 1	Model 2	Model 3
Continuous (ln RC)	1.17 (0.85, 1.62), 0.3305	1.30 (0.90, 1.89), 0.1629	1.33 (0.83, 2.13), 0.2387
Tertile 1	Reference	Reference	Reference
Tertile 2	1.26 (0.81, 1.96), 0.3128	1.33 (0.82, 2.15), 0.2421	1.37 (0.80, 2.35) 0.2552
Tertile 3	1.45 (0.94, 2.22), 0.0936	1.56 (0.97, 2.50), 0.0657	1.55 (0.86, 2.79), 0.1430
*P* for trend	0.0944	0.0670	0.1498
Male			
Continuous (ln RC)	0.63 (0.39, 1.01), 0.0526	0.70 (0.40, 1.21), 0.1965	0.88 (0.43, 1.78), 0.7158
Tertile 1	Reference	Reference	Reference
Tertile 2	0.65 (0.35, 1.19), 0.1643	0.69 (0.35, 1.35), 0.2771	0.77 (0.36, 1.69) 0.5216
Tertile 3	0.68 (0.37, 1.25), 0.2192	0.81 (0.41, 1.58), 0.0129	0.95 (0.40, 2.24) 0.9002
*P* for trend	0.2041	0.5091	0.8943
Female			
Continuous (ln RC)	2.17 (1.36, 3.46), 0.0011	2.37 (1.38, 4.07), 0.0018	2.14 (1.07, 4.27), 0.0304
Tertile 1	Reference	Reference	Reference
Tertile 2	3.33 (1.60, 6.96), 0.0013	3.40 (1.56, 7.43), 0.0021	3.14 (1.32, 7.46), 0.0094
Tertile 3	3.74 (1.81, 7.75), 0.0004	3.67 (1.68, 8.01), 0.0011	3.17 (1.25, 8.04), 0.0151
*P* for trend	0.0005	0.0087	0.0260
*P* for interaction	0.0002	0.0036	0.0042

The analytical models were defined as follows Model 1: unadjusted; Model 2: adjusted for sex, age, and race; and Model 3: sex, age, race, education, BMI, hypertension, diabetes mellitus, smoking status, drinking status, Cr, UC, HDL-C, 25(OH)D, AST, ALT.

**Figure 2:**
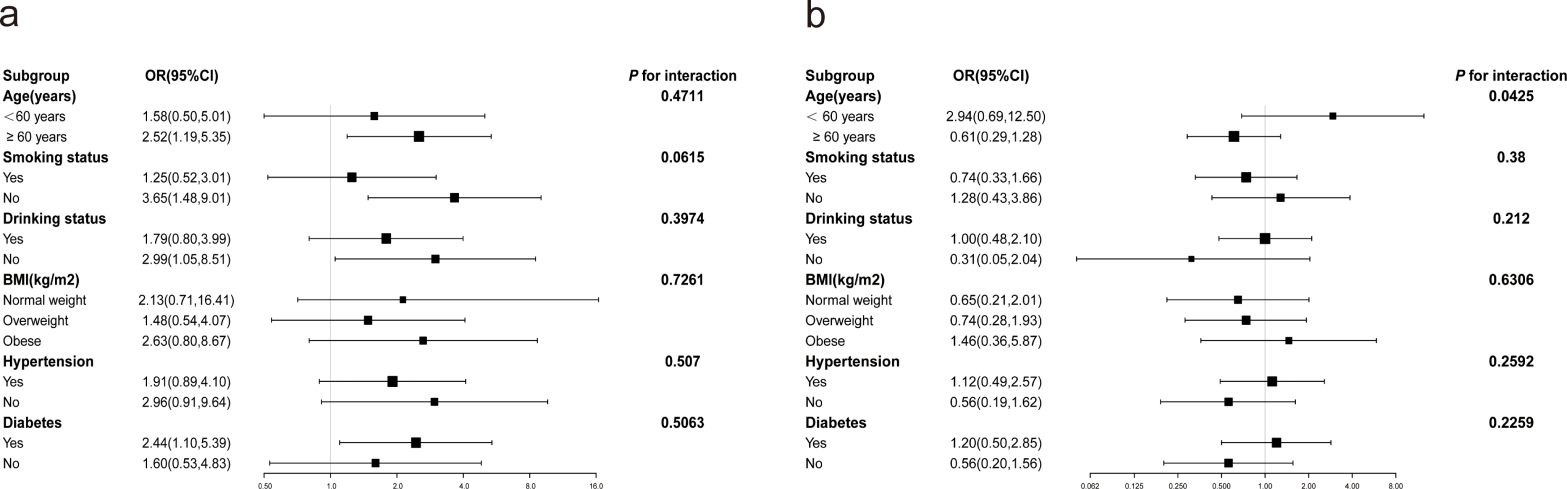
Subgroup analysis of the correlation between RC and severe AAC. Potential moderators of the association between RC and the prevalence of severe abdominal aortic calcification were stratified by sex. **(A)** Females; **(B)** Males. In addition to stratification variables, each subgroup analysis was adjusted for age, race, education, BMI, hypertension, diabetes mellitus, smoking status, drinking status, Cr, UC, HDL-C, 25(OH)D, AST, ALT.

Stratified analyses for male and female participants were separately conducted to evaluate the association between RC and the prevalence of severe AAC across different subgroups (**Figure 2**). In both female (**Figure 2A**) and male (**Figure 2B**) participants, no significant modification of the association between RC and the prevalence of severe AAC was observed with variables such as BMI, smoking status, drinking status, hypertension, and diabetes (*P* for all interactions > 0.05). However, age was found to modify the association between RC and the prevalence of severe AAC in male subjects, a trend not observed in female subjects.

**Figure 3:**
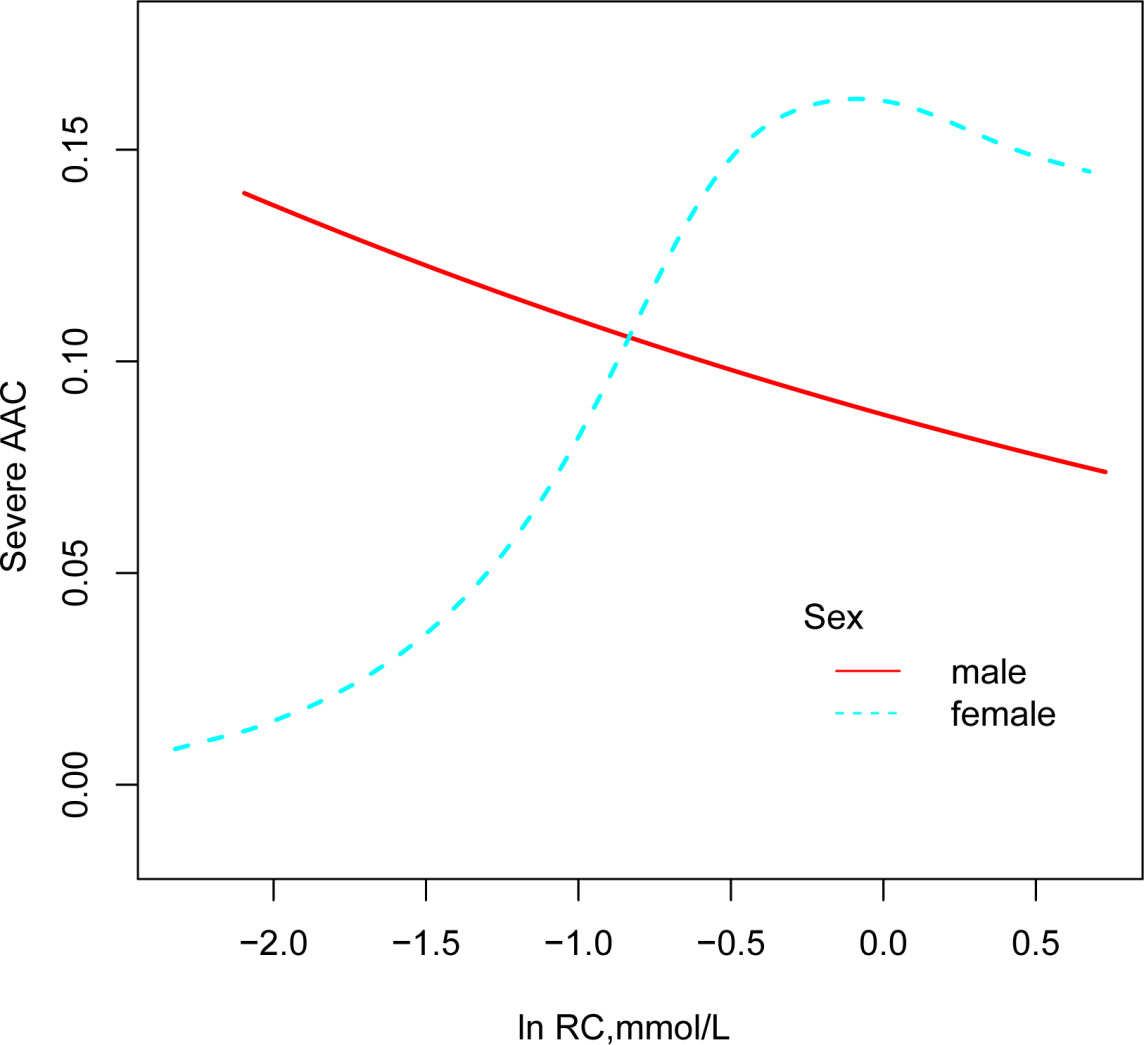
Association between RC and prevalence of severe AAC (by sex). All models were adjusted for sex, age, race, education, BMI, hypertension, diabetes mellitus, smoking status, drinking status, Cr, UC, HDL-C, 25(OH)D, AST, ALT.

Curve fitting techniques were utilized to explore the nonlinear relationship between ln RC and severe AAC, with analyses stratified by gender, as illustrated in **Figure 3**. In female participants, a positive association between ln RC and the prevalence of severe AAC was observed, a pattern not evident in male participants. According to the threshold effect analysis in **Table 3**, this nonlinear relationship was identified exclusively in female subjects (log-likelihood ratio < 0.05). The analysis revealed a saturation effect, indicating a significant 8.19-fold increase in the prevalence of severe AAC for each unit increase in ln RC at values less than -0.56, corresponding to an RC < 0.57mmol/L (OR 8.19, 95% CI 2.06-32.53). However, at ln RC values greater than -0.56 (RC > 0.57 mmol/L), no significant correlation between ln RC and severe AAC was observed (OR 0.61, 95% CI 0.17-2.18).

**Table 3:** Threshold analysis of the effect of ln RC on severe AAC using two-piece linear regression models.

Severe AAC	OR (95%) *P* value	
	male	female
ln RC		
Inflection point	-0.67	-0.56
<Inflection point	0.55 (0.16, 1.93) 0.3493	8.19 (2.06, 32.53) 0.0028
>Inflection point	1.32 (0.42, 4.11) 0.6364	0.61(0.17, 2.18) 0.4472
Log likelihood ratio	0.384	0.014

All models were adjusted for age, race, education, hypertension, diabetes mellitus, smoking status, drinking status, Cr, UC, BMI, HDL-C, 25(OH)D, AST, ALT.

## Discussion

This study is the first to explore the correlation between RC and severe AAC, revealing that gender may play a modifying role in this relationship. A positive correlation between RC and the prevalence of severe AAC was observed in female participants, remaining significant even after adjusting for confounding factors such as race, age, education level, and BMI (per natural log [RC] increment: OR, 2.14; 95% CI 1.07-4.27). However, this relationship was not observed in male participants. In females, smooth curve fitting and threshold effect analysis revealed a non-linear relationship. When ln RC is less than -0.56 (equivalent to RC being less than 0.57mmol/L), each unit increase in ln RC was associated with an 8.19-fold increase in the odds of severe AAC (OR 8.19, 95% CI 2.06-32.53). Conversely, when ln RC is greater than -0.56, no correlation was found between ln RC and severe AAC (OR 0.61, 95% CI 0.17-2.18).

Prior studies have investigated the correlation between AAC and lipid profiles. One such study in a blood dialysis population found no significant link between AAC severity and levels of LDL-C, HDL-C, triglycerides, or lipoprotein (a) (16). However, different results have emerged from other research. For example, the MESA study demonstrated a significant association between elevated TC, reduced HDL-C, and an increased risk of AAC (3). Furthermore, an analysis of 1078 patients without cardiovascular disease revealed correlations between AAC and levels of HDL-C and non-HDL-C, as well as the TC to HDL-C ratio (17).

Despite these findings, the role of lipids in the progression of AAC remains uncertain. Terry JG et al. noted that, despite significant reductions in TC, triglycerides, and LDL-C, simvastatin treatment did not slow the progression of CAC or AAC (18). Similarly, a study involving 16 patients with familial hypercholesterolemia treated with high-dose statins/ezetimibe found little impact of baseline or treatment-level TC or LDL-C on AAC progression (19).

Global guidelines consistently emphasize that LDL-C is a central factor in the development of atherosclerotic cardiovascular disease (ASCVD), positioning it as a central target for prevention and treatment (20, 21). However, even after significant LDL-C reduction to recommended targets with statin therapy, the risk of recurrent cardiovascular events in patients remains higher than expected (9). This suggests the presence of other risk factors beyond LDL-C, HDL-C, hypertriglyceridemia, and lipoprotein (a). RC, including chylomicron remnants produced by the intestines and VLDL and IDL cholesterol from the liver, is transformed into triglyceride-rich remnants in circulation (22). Multiple studies have confirmed the link between RC and cardiovascular risk. For every 1 mmol/L increase in non-fasting RC, the risk of ischemic heart disease increased 2.8-fold, regardless of HDL cholesterol levels (23). In overweight or obese individuals at high risk for heart disease, triglyceride and RC levels, rather than LDL-C, are linked to cardiovascular outcomes, regardless of other risk factors (24). A cohort study in Denmark involving 109,574 individuals found that reducing RC to 0.8 mmol/L in individuals diagnosed with myocardial infarction/ischemic stroke could reduce recurrent major cardiovascular events by 20% in secondary prevention (9). The concentration of RC is an important predictor of cardiovascular disease risk, potentially surpassing that of LDL-C (25). However, the relationship between RC and AAC has not been investigated in prior studies.

This study revealed a significant association between elevated RC levels and increased severe AAC incidence in females, with a noted saturation effect. Subgroup analyses confirmed this stable relationship in the female subgroup. One possible explanation for the positive correlation between RC and severe AAC is that RC, similar to LDL cholesterol, infiltrates and remains in the intima-media layer of the arterial wall, leading to cholesterol accumulation, atherosclerosis, and ultimately severe calcification. Experimental studies have also identified potential mechanisms by which RC are associated with endothelial dysfunction, such as impaired vasodilatation and enhanced inflammatory response, leading to plaque rupture and thrombosis (26).

The results of the interaction test showed that there was a significant gender difference in the risk of severe AAC associated with RC, with female participants having a higher risk. Similarly, this gender difference is observed in the association between RC and conditions such as NAFLD and metabolic syndrome (15, 27). This difference is also reflected in the higher association between RC and mortality in the female subgroup compared to the male subgroup (28). The NHANES study only screened for AAC in people over the age of 40; therefore, this study focused on the middle-aged and older population. Among the baseline characteristics of the study participants, it was found that men with higher RC were younger, whereas women with higher RC were older. Thus, the increased risk of severe AAC in women with high RC may be due to estrogen deficiency in postmenopausal women. Estrogen is closely linked to cholesterol metabolism and plays a cardioprotective role by inhibiting inflammation and atherosclerosis, ameliorating vascular damage, and reducing vascular calcification (29). Studies have found that women who have undergone bilateral salpingo-oophorectomy have a five-fold increased risk of calcification of the abdominal aorta (30). Estrogen replacement therapy greatly reduces the risk of coronary artery or aortic calcification in women (31–33). Given the lack of established normal ranges or measurement standards for RC and the need for further research to clarify its mechanisms, additional studies are urgently needed to confirm our findings and better understand the underlying processes (34, 35). As research on the relationship between RC and AAC advances, therapeutic strategies targeting RC regulation may emerge as a new area of focus.

### Study strengths and limitations

This study has two significant advantages that bolster the credibility and validity of its results. First, the large sample size and the consistency between preliminary and sensitivity analysis findings underscore the robustness of the results. Secondly, RC is easily calculable and obtainable, making it particularly suitable for chronic disease risk assessment and epidemiological surveys in the general population.

However, there are limitations. First, the cross-sectional nature precludes inferences about causality. Second, despite adjustments for a wide range of previously selected confounding factors due to the observational nature of this study, other confounding factors may still exist. Considering these limitations, a carefully designed prospective cohort trial is required to validate our findings.

## Conclusion

This cross-sectional study indicates a positive correlation between RC and severe AAC among American women. RC can be utilized to identify the female population at risk for severe AAC. This highlights the importance of considering gender differences in cardiovascular risk assessments and may guide future research into targeted prevention strategies.

## Data Availability

Publicly available datasets were analyzed in this study. This data can be found here: https://www.cdc.gov/nchs/nhanes.
